# Comparative Analysis of Anther Transcriptome Profiles of Two Different Rice Male Sterile Lines Genotypes under Cold Stress

**DOI:** 10.3390/ijms160511398

**Published:** 2015-05-18

**Authors:** Bin Bai, Jun Wu, Wen-Tao Sheng, Bo Zhou, Li-Jie Zhou, Wen Zhuang, Dong-Ping Yao, Qi-Yun Deng

**Affiliations:** 1 State Key Laboratory of Hybrid Rice, Longping Branch of Graduate School, Central South University, Changsha 410013, China; E-Mails: baibin87@163.com (B.B.); wujun1984@126.com (J.W.); shengwentao2003@163.com (W.-T.S.); zhoulijie2006@163.com (L.-J.Z.); 2 Hunan Hybrid Rice Research Center, Changsha 410125, China; E-Mails: zhuang_wen@163.com (W.Z.); yaodongping1218@126.com (D.-P.Y.); 3 Plant Breeding, Genetics, and Biotechnology Division, International Rice Research Institute, DAPO Box 7777, Metro Manila 1301, Philippines; E-Mail: b.zhou@irri.org; 4 College of Agronomy, Hunan Agricultural University, Changsha 410128, China

**Keywords:** cold stress, transcriptome, RNA-Seq, anther, PTGMS rice

## Abstract

Rice is highly sensitive to cold stress during reproductive developmental stages, and little is known about the mechanisms of cold responses in rice anther. Using the HiSeq™ 2000 sequencing platform, the anther transcriptome of photo thermo sensitive genic male sterile lines (PTGMS) rice Y58S and P64S (Pei’ai64S) were analyzed at the fertility sensitive stage under cold stress. Approximately 243 million clean reads were obtained from four libraries and aligned against the oryza indica genome and 1497 and 5652 differentially expressed genes (DEGs) were identified in P64S and Y58S, respectively. Both gene ontology (GO) and Kyoto Encyclopedia of Genes and Genomes (KEGG) analyses were conducted for these DEGs. Functional classification of DEGs was also carried out. The DEGs common to both genotypes were mainly involved in signal transduction, metabolism, transport, and transcriptional regulation. Most of the DEGs were unique for each comparison group. We observed that there were more differentially expressed MYB (Myeloblastosis) and zinc finger family transcription factors and signal transduction components such as calmodulin/calcium dependent protein kinases in the Y58S comparison group. It was also found that ribosome-related DEGs may play key roles in cold stress signal transduction. These results presented here would be particularly useful for further studies on investigating the molecular mechanisms of rice responses to cold stress.

## 1. Introduction

Nearly half of the world population depends on rice as a staple food. Thus, increasing rice yields to help meet and ensure world food security is a significant and pressing technological goal. Cold stress is one of the major constraints in rice production and productivity, especially in temperate and subtropical zones and in high-altitude areas. Cold stress affects both the vegetative and the reproductive phases of the rice life cycle, with the latter being particularly sensitive to low temperatures [1]. Low temperatures conditions during the reproductive growth stages of rice often cause abnormal development of anthers, which eventually results in low spikelet fertility and significant yield losses. The expression of a number of genes has been reported to be induced by cold; the gene products of such genes are assumed to function in cold stress tolerance and signaling responses [2]. Genome-wide gene profiling using microarray technologies has revealed complex regulatory networks active in cold tolerance responses in plants. It is well known that the dehydration-responsive element binding protein (DREB1)/C-repeat binding factor (CBF) cold-responsive pathway is conserved in rice and this is a key regulatory pathway in plants [3,4]. Using cDNA microarray analysis, 64 genes were identified as candidate cold-inducible genes when rice seedlings were exposed to cold for 5, 10, or 24 h. The products of these genes were classified into two groups: one group consisting of function proteins such as late embryogenesis-abundant (LEA) proteins, cold-acclimation proteins, carbohydrate metabolism-related proteins; the other group consisting of various types of transcription factors such as Myeloblastosis (MYB), NAM-ATAF-CUC (NAC), and basic region/Leu zipper motif (bZIP) family transcription factors [5]. Transcriptome analysis using a cDNA microarray in chilling-tolerant *japonica* rice indentified 121 genes that were induced by 24 h of treatment at 10 °C, and found that a ROS (reactive oxygen species)-bZIP1 regulon makes an important contribution in early responses to chilling stress [6]. Further genome-wide gene profiling revealed that the expression of several H_2_O_2_-induced classes of bZIP factors, ethylene-responsive factors (ERF), and R2R3-MYB factors could be induced in early chilling responses [7]. Through comparing the transcriptomes of cold-tolerance gene OsNAC5-over-expressing transgenic plants and control plants, or cold-tolerant genotype Li–Jiang–Xin–Tuan–Hei–Gu (LTH) and cold-sensitive genotype IR29, the expression of many stress-inducible genes was observed up-regulated in the cold-tolerant genotype in response to cold treatment [8,9].

Previous transcriptome studies of cold responses in rice were performed using only the leaves of rice at the seedling stage, ignoring the relatively more cold-sensitive booting stage and further ignoring gene expression in organs that are known to be highly related to yield, such as anthers. It is imperative to study the genetic mechanisms of low temperature tolerance at the booting stage, in light of the paramount significance of this stage in rice cultivation. Microarray technologies have drawbacks, such as high background signal and pre-determined probe identities [10,11]. RNA sequencing technology (RNA-Seq) is an ideal method for transcriptomic analysis due to its high sensitivity, low background noise, and the fact that it enables one to distinguish different isoforms and allelic expression [12]. RNA-Seq has been used for transcriptional analyses in rice over the last three years [13,14,15,16,17,18,19].

Two-line hybrid rice breeding has made great progress in China. Up to 2012, the total planting area of two-line hybrid rice in China reached 31.67 million hectares, and this technology has great potential to spread throughout the whole world [20]. Photo-thermo sensitive genic male sterile lines (PTGMS) are the basis of two-line hybrid rice breeding technology. Having relatively lower temperatures that will induce “fertility alternation” during the fertility sensitive stage is a key technical requirement for the use of PTGMS lines in the production of hybrid rice seeds [21]. However, low temperature (temperatures less than 20 °C) pose the risk of physiological damage to PTGMS lines, and can lead eventually to a yield loss. Therefore, in breeding programs, it is highly desirable to have a PTGMS line that has both a low critical temperature for the induction of fertility alteration and a robust physiological tolerance to low temperatures. The study of the effects of cold stress on reproductive development in rice has been reviewed [22,23], but the molecular mechanisms underlying cold tolerance in rice PTGMS lines has not been reported previously. The study of the molecular mechanisms through which these lines respond to low temperatures is of fundamental importance in rice biology.

In this study, RNA-Seq was used to investigate two different PTGMS lines under both fertility temperatures (22 °C for 10 days) and cold treatment conditions (17.5 °C for 10 days) at the booting stage. Differentially expressed genes were identified from these data. This study of differentially expressed genes following cold stress treatment furthers our understanding of the cold response mechanisms of rice and contributes to the technological resources available for the improvement of cold tolerance in rice PTGMS lines.

## 2. Results

### 2.1. The Differences in Seed Setting Rates of Two Rice Photo-Thermo Sensitive Genic Male Sterile Lines (PTGMS) Following Cold Stress during the Fertility Sensitive Stage

**Figure 1:**
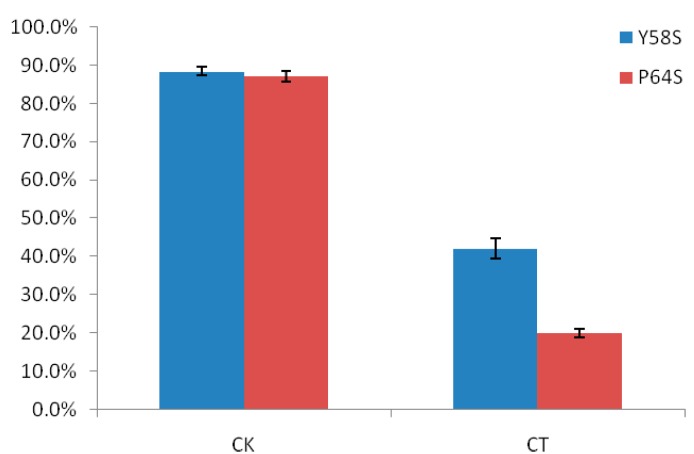
The comparison of the seed setting rates of Y58S and P64S in the control and in the cold treatment. CK indicates control treatment; CT indicates cold treatment. Vertical bar indicates standard error.

The differences in seed setting rates between the rice PTGMS lines Y58S and Pei’ai64S (P64S) are shown in Figure 1. In the control experiment, the seed setting rates of Y58S and P64S were 88.4% and 87.1%, respectively. There was no obvious difference between these two varieties. However, the seed setting rates of Y58S and P64S following cold treated experiment were very different: the seed setting rate of Y58S was 42.0% while the rate of P64S was only 20.0%, the difference in seed setting rates between these two varieties following cold treatment was statistically significant. From these data, we can conclude that the Y58S genotype was more cold tolerant than P64S at the fertility sensitive stage following cold stress treatment.

### 2.2. RNA Sequencing Technology (RNA-Seq) and Read Assembly

Four RNA samples were prepared from anthers collected from the four experimental conditions (control and cold-treated Y58S, control and cold-treated P64S). These samples were sequenced using the Illumina HiSeq™ 2000 platform. A total of about 260 million raw sequencing reads with lengths of 100 bp were generated (Table 1). We discarded low-quality reads including adapters and unknown or low-quality bases, according to our bioinformatics analysis. Finally, we obtained about 243 million clean reads, representing 93.3% of the total raw reads. The average Q20, Q30, and GC contents were 95.96%, 89.22%, and 52.11%, respectively. Two biological replicates were evaluated for the cold stress-treated and the control samples; the results for the two replicates were found to be in good agreement, with 0.975 < *R*^2^ < 0.983 (Figure S1). Clean reads were then mapped to the Oryza indica genome using Tophat v2.0.9. Of the total clean reads from the four sample groups, 85.18%–86.6% were uniquely mapped, and 2.32%–2.56% were mapped to multiple loci; 10.75%–12.29% of reads were unmapped (Table 1).

**Table 1:** Summary of sequencing data quality and the statistics of the transcriptome assembly.

Sample	CP ^a^	CY ^b^	TP ^c^	TY ^d^
Raw reads	47,121,266	69,710,112	69,494,844	73,760,444
Clean reads	44,065,042	65,064,778	64,395,762	69,199,314
Clean bases	4.4 G	6.50 G	6.44 G	6.92 G
Q20 (%)	96.04	95.91	95.57	96.31
Q30 (%)	89.42	89.15	88.33	90.00
GC content (%)	52.45	52.79	52.20	51.01
Total mapped	39,081,161 (88.69%)	57,550,416 (88.45%)	56,480,119 (87.71%)	61,762,180 (89.25%)
Multiple mapped	1,068,940 (2.43%)	1,512,396 (2.32%)	1,626,645 (2.53%)	1,835,764 (2.65%)
Uniquely mapped	38,012,221 (86.26%)	56,038,020 (86.13%)	54,853,474 (85.18%)	59,926,416 (86.6%)

^a^ CP indicates control treated P64S; ^b^ CY indicates control treated Y58S; ^c^ TP indicates cold treated P64S; ^d^ TY indicates cold treated Y58S.

### 2.3. Identification of Differentially Expressed Genes Following Cold Stress

**Figure 2:**
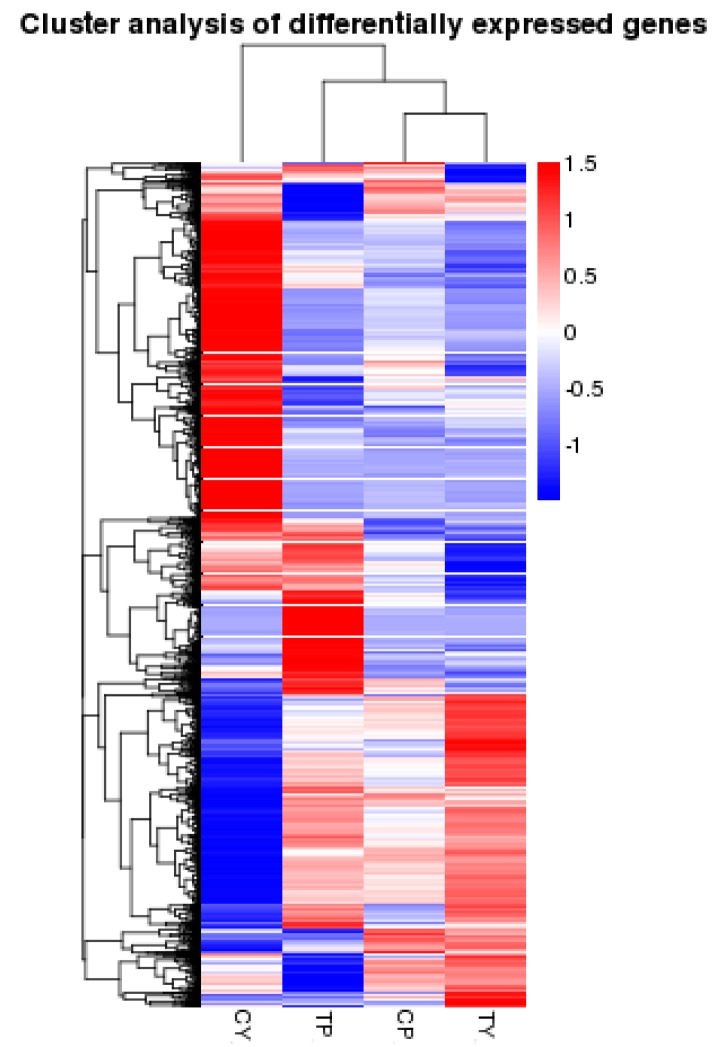
Hierarchical cluster analysis of gene expression based on the log ratio fold change data. The color scale indicates the gene expression level: red color represents increased transcript abundance and blue color represents decreased transcript abundance. TP and CP indicate cold treated P64S and control treated P64S, respectively. TY and CY indicate cold treated Y58S and control treated Y58S, respectively.

Genes with similar expression patterns often have similar functions, or are involved in the same metabolic processes or pathways. Thus, clustering of genes with similar expression patterns is an analytical strategy that can contribute to the identification of the function of unknown genes or can help to characterize the unknown functions of known genes. To identify clusters with functional enrichment, we performed hierarchical clustering based on gene expression patterns (Figure 2). We found that gene expression in P64S plants showed only small differences following cold stress treatment, while there were large differences in gene expression in Y58S plants. Gene expression levels were calculated using the reads per kb per million reads (RPKM) values. Differentially expressed genes (DEGs) between sample groups were defined using fold change values of the normalized (RPKM) expression values. We filtered DEGs with |log2 (Fold Change)| > 1 and corrected *p*-value (padj) < 0.05; 1497 DEGs were thusly identified between the control and cold-treated P64S samples (Table S1), and 5652 DEGs were identified between the control and cold-treated Y58S samples (Table S2).

### 2.4. Functional Classification of Genes Induced by Cold Treatment Using Gene Ontology (GO) and Kyoto Encyclopedia of Genes and Genomes (KEGG) Analysis

**Figure 3:**
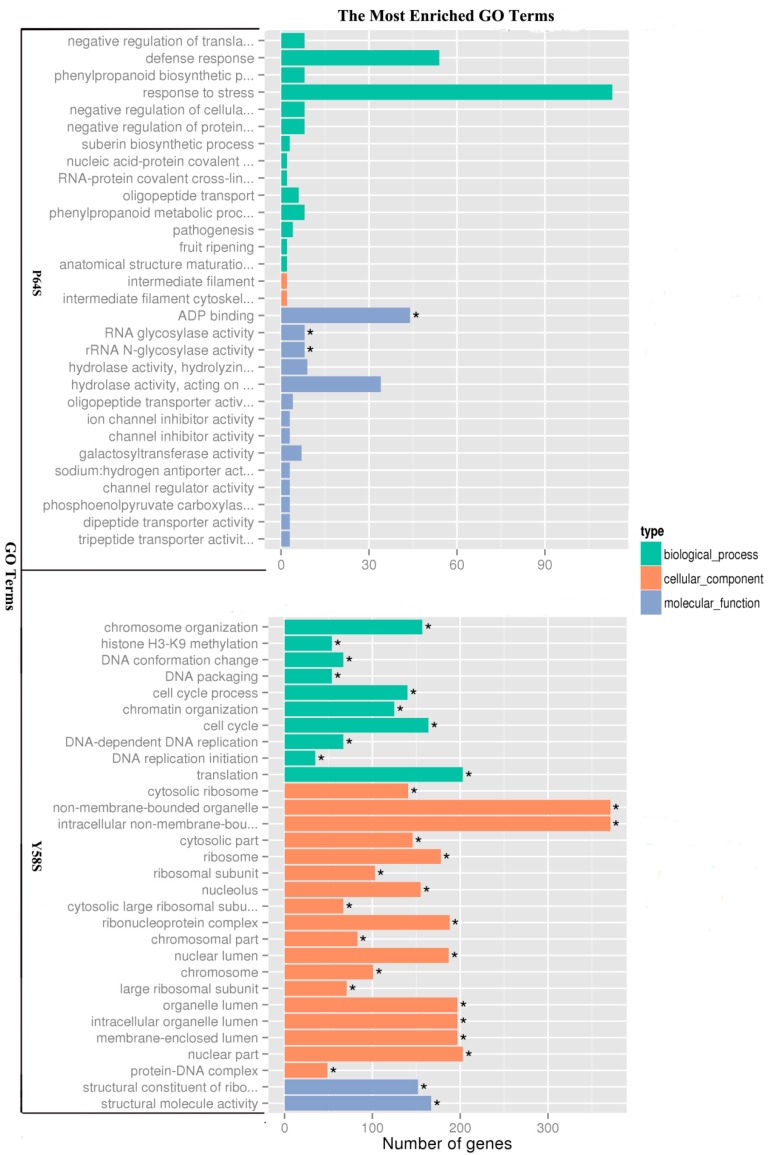
Gene ontology (GO) classifications of differentially expressed genes (DEGs) in P64S and Y58S, respectively. * indicates significantly enriched GO terms with correct *p*-value < 0.05.

The gene ontology (GO) assignment system was used to obtain functional information for the DEGs, a procedure that can assist in understanding the distribution of gene functions at a macro level. All unigenes were then assigned into three main GO categories: biological process, cellular component, and molecular function. GO analysis of the up-regulated and down-regulated genes in the P64S and Y58S plants are shown in Figure 3. In the P64S comparison, the 1497 DEGs were enriched in 2007 functional terms. “Response to stress” (GO: 0033554), “defense response” (GO: 0006952), “ADP binding” (GO: 0043531), and “hydrolase activity, acting on glycosyl bonds” (GO: 0016798) were the four most highly enriched GO terms. A GO term was considered to be significantly enriched if the corrected *p*-value was below 0.05. Significantly enriched GO terms included “ADP binding” (GO: 0043531), “RNA glycosylase activity” (GO: 0030597), and “rRNA *N*-glycosylase activity” (GO: 0030598) in P64S.

There were more significantly enriched GO terms in the Y58S plants than in the P64S plants. “Non-membrane-bounded organelle” (GO: 0043228) and “intracellular non-membrane-bounded organelle” (GO: 0043232) were the two most highly enriched terms.

KEGG (Kyoto Encyclopedia of Genes and Genomes) pathway analysis provides classifications that are valuable for studying the complex biological functions of genes. KEGG analysis showed that “carotenoid biosynthesis” (ko00906) was significantly enriched in the P64S DEGs (Table 2). For the Y58S DEGs, the significantly enriched KEGG pathways were “Ribosome” (ko03010), “DNA replication” (ko03030), “Cutin, suberine and wax biosynthesis” (ko00073), and “Galactose metabolism” (ko00052).

**Table 2:** The significantly enriched pathway terms in the RNA-Seq analysis.

Term	Sample Number	Background Number	Corrected *p*-Value	Comparison
Carotenoid biosynthesis	6	24	0.03071	P64S
Ribosome	122	323	5.45 × 10−9	Y58S
DNA replication	24	54	0.00930	Y58S
Cutin, suberine and wax biosynthesis	10	15	0.00930	Y58S
Galactose metabolism	21	46	0.00930	Y58S

### 2.5. Cold Response Genes Common to P64S and Y58S

Following cold treatments, 304 DEGs were identified in both P64S and Y58S plants (Table S3). Signal transduction, metabolism, transport, and transcription factor (TF) genes were found among the up-regulated DEGs that were common to both genotypes. These genes encoded products such as calcium/calmodulin dependent protein kinases, receptor protein kinases, and transporter proteins that are known to be key components in calcium signal transduction. Metabolism-related genes relating to lignin biosynthesis and fatty acid synthesis were also found among the DEGs common to both genotypes. Eight genes for retrotransposons and three genes for transposons were identified among the up-regulated genes common to both genotypes. Three zinc finger TF genes and one APETALA2 (AP2) TF gene were found to be up-regulated following cold stress treatment. In addition, two zinc finger TF genes (*LOC_Os01g50750* and *LOC_Os05g44550*) had down-regulated expression following cold stress treatment. Three MYB family TF genes were also found to be down-regulated in both genotypes. Down-regulated genes mainly included cyclins, cytochrome P450s, aquaporins, peroxidase precursors, and phospholipases.

### 2.6. Difference of Cold Response Differentially Expressed Genes (DEGs) in P64S and Y58S

In total, 5652 and 1497 DEGs were identified in Y58S and P64S in the cold stress experiments, respectively; 2221 up-regulated DEGs and 3431 down-regulated DEGs were detected in Y58S. In P64S, there were 720 up-regulated and 777 down-regulated DEGs (Tables S1 and S2). The TF genes identified among the DEGs mainly belonged to the AP2/ERF, bHLH (basic helix-loop-helix proteins), MYB, bZIP, WRKY, and zinc finger families (Table S4). Twenty-two AP2/ERF TF genes were identified as cold responsive (COR) genes in Y58S; only six of these were up-regulated during cold stress. The largest TF group was the zinc finger family; there were 126 and 26 zinc finger family genes in Y58S and P64S, respectively.

Seven light-responsive genes were specified as COR genes in Y58S, of which three genes encoding light-induced chloroplast precursors were up-regulated by cold stress treatment (Table S5). Downstream events of many signal molecules are mediated through various protein kinases. The COR protein kinases mainly consisted of members from the LRR (leucine-rich repeat), Ser/Thr protein kinase, and calcium-related kinases families. Calmodulin-related genes and plant hormone signal transduction genes were also identified as COR genes. COR genes encoding a diverse array of proteins such as enzymes involved in the metabolism of carbohydrates, lipids, antioxidants, and plant hormones were also identified (Tables S5 and S6). Subsequent functional analysis of these stress-inducible genes should foster a deeper understanding of the complex regulatory network involved in rice anther responses to cold stress.

### 2.7. DEGs Mapped to the Previously Identified Quantitative Trait Loci (QTLs) Controlling Cold Tolerance at the Reproductive Stage

A total of nine QTLs (quantitative trait loci) related to cold tolerance in rice have been identified, according to the Gramene QTL database (http://www.gramene.org/). We located 220 DEGs on these QTL intervals. Among them, the QTLs qCTB1, qPSST-9 and LTH had the greatest number of co-localized DEGs with 37, 29, and 32 genes, respectively (Table S7).

A QTL for cold tolerance at the booting stage, qCTB1, was fine-mapped [24]. Twenty genes on this interval were found to be differentially regulated by cold stress in this study. Three genes encoding zinc finger proteins were strongly repressed exclusively in Y58S, while a gene encoding chlorophyll a–b binding protein was highly induced in Y58S. The QTL LTH was recently identified by using F_2_ and BC_1_F_2_ populations [25]. Thirty-two genes were co-localized on this interval. Among them, four zinc finger proteins, two phosphatases, two transferases and two ribosomal proteins were evidently induced by cold stress in either Y58S or P64S. By combining QTL mapping and functional analysis, the co-localized DEGs detected in this RNA-Seq data may provide more information for gene cloning and elucidation of the molecular mechanisms involved in cold stress responses.

### 2.8. Validation of the Gene Expression Profiles by qRT-PCR

**Figure 4:**
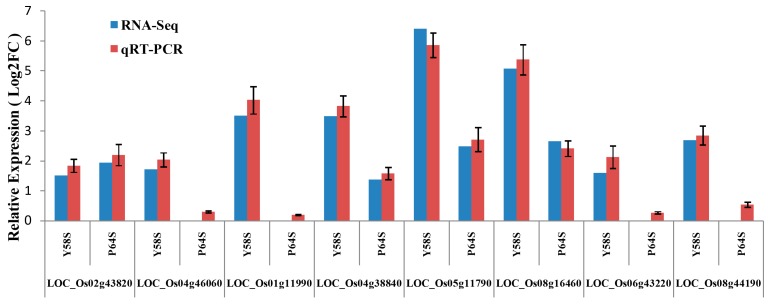
Comparison of genes expression levels using RNA-Seq and qRT-PCR. The relative expression values were normalized to the rice actin 1 gene. Error bars indicate standard deviation. Log2FC refers to the log2 fold change.

To validate the expression profiles of the genes in our transcriptome sequencing analysis, eight genes with significantly altered expression following cold stress treatment were evaluated with qRT-PCR, all of which are predicted to be related to cold stress, functional annotations of these genes are listed in Table S8. The expression patterns of all eight genes were the same in the qRT-PCR analysis as they were in the RNA-Seq analysis, these results are shown in Figure 4.

## 3. Discussion

In this study, we used RNA-Seq analysis to evaluate the transcriptomes of the anthers of two different PTGMS rice genotypes at the fertility sensitive stage following cold stress treatment. Y58S, developed by crossing among four genotypes including P64S, is known to have wide ecological adaptability and strong stress resistance [26]. Although cold stress tolerant rice breeding programs have made some progress, the molecular and genetic mechanisms underlying cold responses remain poorly understood [27,28]. In our RNA-Seq analysis, 260 million raw reads with lengths of 100 bp were generated. 5652 DEGs in Y58S and 1497 DEGs in P64S were identified. These results suggest that in the cold stress tolerant lines, more COR genes may be involved in regulatory network, than in cold stress sensitive lines. KEGG and protein function analysis revealed that most of the unigenes of DEGs were annotated to specific pathways, such as metabolic pathways, biosynthesis of secondary metabolites, ribosome, carbon metabolism, and plant hormone signal transduction.

### 3.1. The Role of Transcription Factors in Rice Responses to Cold Stress

The key of plant tolerance to cold stress is a complex regulatory network involving transcription factors and other regulatory genes that control enzymes, regulatory proteins, and metabolites [29]. In this study, genes from six TF families including the AP2/ERF, bHLH, MYB, bZIP, WRKY, and zinc finger families were identified as COR genes (Table S4). Cold stress induced the expression of AP2/ERF family TFs, which can then activate the expression of COR genes, this pathway plays a crucial role in gene regulation during cold stress [30]. Twenty-two AP2/ERF genes were identified as DEGs in our comparisons, suggesting that they play important roles in rice responses to cold stress. ICE1, a bHLH protein in *Arabidopsis*, is known to bind specially to the promoter region of *CBF3* (C-repeat binding factors 3) and increases the expression of CBF3, which in turn leads to enhanced tolerance to cold [31]. Among the 13 COR bHLH genes identified in our study, *LOC_Os07g36460* (Udt1), *LOC_Os04g51070* (EAT1), and *LOC_Os02g02820* (TDR) are known to be involved in pollen formation and anther development, TDR can interact Udt1 and EAT1 to regulate the early development of rice anther [32]. Our results indicate that cold stress affects anther development in rice and we conclude that *bHLH* genes play important roles in this process. In *Arabidopsis*, another known direct regulator of *CBF3* is MYB15, which can bind to the promoter region of *CBF3* and repress its expression. ICE1 can interact with MYB15 and attenuate its expression in response to cold in wheat [33]. *LOC_Os01g62410* (OsMYB3R-2), identified as an up-regulated gene in Y58S, encodes a MYB activation TF that is known to increase cold tolerance by regulating the CBF pathway and cell cycle progression during cold stress [34]. MYBS3 (*LOC_Os10g41200*) is known to regulate a cold signaling pathway [35]. These data suggested that MYB family members may be good candidates for improving rice cold tolerance.

A number of bZIP TFs are known to function in stress signaling in plants, but few of these have been characterized in rice. Ten COR bZIP genes were identified in Y58S, and one was identified in P64S. Two of these, OsABF1 (*LOC_Os01g64730*) and OsbZIP52 (*LOC_Os06g45140*), were previously characterized and found to be involved in cold stress responses [36,37]. WRKY TFs have been reported to be involved in diverse signaling pathways; these proteins often act as activators as well as repressors [38]. Sixteen WRKY genes have been reported to play important roles in the regulation of cold stress in rice, and the expression of most of them is known to be down-regulated by cold stress treatment [39]. Our results were consistent with this trend for WRKY TFs. Zinc finger TFs were the largest group of TFs found to be differentially expressed in our data; 152 zinc finger proteins were induced following cold stress treatment. The expression of OsiSAP8 (*LOC_Os06g41010*) has been shown to be induced by cold stress [40]. The expression of several genes, such as OsTZF1 (*LOC_Os05g10670*) and OsDOS (*LOC_Os01g09620*), have been found to be induced by drought and heat stress, though not yet by cold stress [41,42]. The expression changes of these genes in our data indicate that they may also be involved in cold stress responses.

RNA helicases might play important roles in regulating stress-induced pathways [2]. A mutation in a DEAD box RNA helicase gene, *los4*, is known to inhibit CBFs regulation during cold stress and to render *Arabidopsis* cold sensitive [43]. Five DEAD-box ATP-dependent RNA helicases and three ATP-dependent RNA helicases were identified as DEGs in our study (Table S4). These RNA helicases may be involved in temperature sensing and responses to changes in temperature.

### 3.2. Cold Signal Transduction

Plant cells can sense cold stress through physical phase transitions in membranes, as well as through changes in protein and nucleic acid conformations. Ca^2+^ acts as a secondary messenger, increases of cold-induced Ca^2+^ concentrations in the cytosol can be mediated through membrane Ca^2+^ channels, which giving rise to calcium signal amplification during cold stress [44]. CDPK13 is known to be a key component in responses to cold stress in rice [45]. Sixteen calmodulin/calcium dependent protein kinase DEGs were identified in our data. Other groups of Ca^2+^ sensors, such as CaM (calmodulin) and CMLs (CaM-related) were also among the DEGs of our study (Table S5). Exploring their roles in rice cold stress responses may be helpful for elucidating the mechanisms of cold tolerance.

One up-regulated DEG in P64S (Table S5), OsAREB1 (*LOC_Os06g10880*), is known to function as a transcriptional regulator that modulates ABA (abscisic acid)-dependent gene expression during abiotic stress [36]. The SnRK2 protein kinase (SAPK) family is known to be involved in distinct regulatory pathways, including ABA responsiveness [46]. Three SAPK genes (*SAPK1*, *SAPK3*, and *SAPK5*) were among the down-regulated DEGs in our data. Although the knowledge of the role of auxin under cold stress is limited, recent experiments suggest that cold stress induced changes in plants are tightly linked to auxin responses. Some important regulatory components of cold signaling pathways have been shown to regulate growth and development through the control of auxin signaling in *Arabidopsis* [47,48]. One bZIP transcriptional factor in rice, ABI5-Like1 (ABL1), is involved in ABA-dependent cold signaling and suppresses auxin signaling, indicating that multiple hormones might be integrated in regulating the cold stress signals [49]. Eighteen auxin-related DEGs were found in our data; these genes might play key roles in cold stress signal transduction (Table S5).

It is known that the fertility of the major rice two-line sterile lines is affected by both light and temperature, so it is possible that the perception of light is involved in cold response. In *Arabidopsis*, exposure of leaves of cold tolerant plants to high light stress in cold stress conditions resulted in decreased photochemical efficiencies in both Photosystem II and Photosystem I, as compared to cold sensitive plants [50]. It has also been reported that the expression of some F-box proteins can be influenced by light and abiotic stresses in rice [51]. Further investigation of the seven light-induced genes identified in our study might help reveal the relationship between light and temperature in plant abiotic stress responses (Table S5).

### 3.3. The Complex Network of Molecular Responses to Cold Stress in Rice

Cold responses are very complex traits that are known to involve many signal pathways, transcriptional regulators, and metabolites [52]. Cell wall kinases, phospholipase D (PLD), and glycoproteins have been proposed to be involved in sensing low temperature [53]. DEGs encoding these proteins or related-proteins were identified in our study (Table S1 and S2). Plant cells are known to sense cold stress through rigidification and physical phase transitions of membranes, which lead to actin cytoskeletal rearrangements and changes in cytosolic Ca^2+^ concentrations. Ca^2+^ then acts as a secondary messenger to induce the expression of cold-responsive genes. ROS and ABA are known to act as signaling molecules to activate downstream signal cascades through the induction of specific Ca^2+^ signatures [2]. Transcriptional cascades then operate through ABA-dependent and ABA-independent pathways to induce the expression of many cold-regulated genes, resulting in increases in the concentrations of metabolites [53]. Some such metabolites confer protective effects against damage, others such as reactive oxygen species can act as signaling molecules. Soluble sugar can also act as a signal to modulate plant growth and development during cold stress. Twenty galactose metabolism-related DEGs were identified in Y58S in this study (Table S6).

The role of DNA replication related genes in abiotic stress tolerance in rice is not well understood. It has been reported that the expression of the *MCM6* (minichromosome maintenance 6) gene was induced in pea seedlings in response to cold stress, and that overexpression of MCM6 promotes salinity stress tolerance without affecting yield [54]. The expression of another gene, *PDH47* (pea DNA helicase 47), is also known to be induced by cold stress [55]. These findings suggest that the overexpression of DNA replication related genes may help in stress tolerance in crop plants. In our data, 26 DNA replication related genes were identified, most of them were up-regulated in Y58S (Table S6). The expression of one of these genes, OsRPA2 (*LOC_Os02g58220*), which encodes a heterotrimeric, single-stranded DNA-binding protein that functions in DNA metabolism, is known to be induced by ultraviolet light exposure [56]. Further study of these DEGs involved in DNA replication might help us elucidate how DNA replication is affected by cold stress.

The KEGG pathway with the largest number of significantly enriched DEGs in our study was “ribosome”; there were 150 such DEGs in Y58S (Table S6). To date, it is not known how ribosome proteins may be involved in responses to abiotic stresses. The main function of ribosomes is the syntheses of cellular peptides, though it is known that some ribosome proteins also participate in developmental processes [57]. Through evaluating changes in the nuclear proteome in response to cold treatment, a 60S acidic ribosomal protein (P2-A) was found to be induced in *Arabidopsis* during cold stress. P2-A is crucial in the elongation step of protein synthesis [58]. Using RNA-Seq analysis, 3 and 81 ribosome DEGs were identified under Pi and Fe deficiency treatments, respectively, revealing that the expression of genes encoding ribosome proteins was regulated in response to abiotic stress in *Arabidopsis* [59]. Two ribosome genes in our data, UbL401 (*LOC_Os09g27930*) and UbL402 (*LOC_Os03g15370*), which encode ubiquitin fusion protein, were recently reported in the literature, failure to process their mRNA resulted in excess levels of UbL40 transcripts, and this situation may have led to abnormal male development. Furthermore, processing their mRNA was shown to be regulated by temperature [60]. Further studies about the ribosome DEGs identified in this study may help uncover new mechanisms in which plants respond to cold stress.

Though the transcriptome data of rice anthers under cold stress were rare at present, the DEGs from this experiment were also conducted to compare with other cold responsive genes identified by microarray hybridization and RNA-Seq technologies. Twenty-four genes differentially expressed by cold stress in Y58S have been found to be regulated by chilling in chilling-tolerant *japonica* rice [6]. Three MYB DEGs (*LOC_Os11g45740*, *LOC_Os01g62410*, and *LOC_Os09g36730*) in our data not only can be induced in early chilling responses of *japonica* rice, but also were observed up-regulated in the cold-tolerant genotype LTH [7,9]; 307 DEGs in Y58S could also be induced in *japonica* cultivar Nipponbare during cold stress [19]. All of these results suggest that these DEGs can be considered as candidate genes related to cold tolerance. By combining previously identified cold stress-related genes and fine-mapping QTLs, the co-localized DEGs detected in this RNA-Seq data can be beneficial for gene cloning and dissecting molecular mechanisms of cold tolerance in rice.

In conclusion, a larger number of cold responsive differentially expressed genes were observed in the Y58S genotype as compared to P64S. The transcriptome analysis presented in this study expands our understanding of plant cold stress responses by identifying differentially expressed genes such as transcription factors, signal transduction components, and genes involved in metabolism. DEGs related to DNA replication and ribosomes were also significantly enriched in our data, implying that these processes may play important roles in responses to cold stress in rice anthers. This survey of differentially expressed genes furthers our understanding of the molecular mechanisms involved in cold stress responses and provides a valuable knowledge base from which to design further studies.

## 4. Experimental Section

### 4.1. Plant Materials and Cold Treatments

The rice PTGMS lines Y58S and Pei’ai64S (P64S) were cultivated in the experimental fields of Hunan Hybrid Rice Research Center until reaching stage five of anther development in the evaluation system proposed by Zhang *et al.* [61]. Stage 5 in this system is characterized by the emergence of the flag leaf. Half of the plants were then moved into an irrigation pond and kept at 22 °C with natural light and humidity for 10 days as the control group. The other half of the plants were moved into a growth chamber (SANYO, Osaka, Japan) as the cold treatment group and kept at 17.5 °C with a 12 h light/12 h dark photoperiod and 80% relative humidity for 10 days, ensuring the illumination, humidity values and other environment factors in the growth chamber close to the natural condition as far as possible. The reproductive growth of individual plants remained well synchronized in both experimental groups. Each of these treatments was repeated three times. In order to compare the cold tolerance differences between the Y58S and P64S PTGMS lines, both lines were grown in the same experimental fields. Spikelet fertility is an effective and reliable indicator to evaluate differences in rice cold tolerance [62]. After temperature treatment and natural ripening, these experimental materials were used for the analysis of their seed setting rates for cold tolerance evaluation. Analysis of variance was performed with Sigmaplot 12.5 software (Systat Software, Inc., San Jose, CA, USA), Duncan’s multiple range test was conducted to compare the mean differences at *p* < 0.05.

### 4.2. Pollen Collection and RNA Isolation

During the cold treatment, the anthers of the Y58S and P64S PTGMS lines developed from the microspore mother cell formation stage to the meiosis stage. Using DAPI (4',6-diamidino-2-phenylindole) staining and fluorescence microscopy, we confirmed that 3–5 mm spikes were nearly at stage 8–9 of anther development, when free microspores are released from the tetrads with the degradation of the callose wall at stage 8b according to Zhang *et al.* [61]. Anthers of 3–5 mm florets were collected from plants for RNA isolation when the distance between the pulvinus of the flag leaf and the pulvinus of the next lower leaf was ±1 cm. To avoid the morphological nonsynchronicity, spikes were collected at the same intervals for each panicle. Total RNA was isolated from anthers using a MiniBEST Universal RNA Extraction Kit (TaKaRa, Otsu, Japan) according to the manufacturer’s instructions. RNA purity was checked with an NanoPhotometer^®^ spectrophotometer (IMPLEN, Munich, Germany) and RNA integrity was assessed with an Agilent 2100 Bioanalyzer (Agilent, Palo Alto, CA, USA).

### 4.3. Library Preparation and Transcriptome Sequencing

A total of three μg of RNA per sample was used as the input material for the RNA sample preparations. These high quality RNA samples were sent to Beijing Novogene Bioinformatics Technology Co., Ltd. (Beijing, China). The sequencing libraries were constructed using a NEBNext^®^ Ultra™ RNA Library Prep Kit for Illumina^®^ (NEB, Ipswich, MA, USA) following the manufacturer’s recommendations, and index codes were added to attribute sequences to each sample. Quality of these libraries was assessed with an Agilent 2100 Bioanalyzer system. The clustering of the index-coded samples was performed with a cBot Cluster Generation System using a TruSeq PE Cluster Kit v3-cBot-HS (Illumina, San Diego, CA, USA) according to the manufacturer’s instructions. The libraries were sequenced using an Illumina HiSeq™ 2000 platform and 100 bp paired-end reads were generated.

### 4.4. Quality Control and Reads Mapping

The fastq format raw reads were deposited to NCBI (accessions: SRP057498), then these reads were processed with in-house Perl scripts. By removing reads containing adapters, ploy-N, and low quality reads from the raw data, clean reads were obtained. Q20 (The percentage of bases with a Phred value >20), Q30 (The percentage of bases with a Phred value >20), and GC (base G and C) content of the clean data were calculated. All downstream analyses were based on clean, high quality data. The reference genome and gene model annotation files were downloaded from the rice genome website (ftp://ftp.ensemblgenomes.org/pub/release-20/plants/fasta/oryza_indica/dna/). An index of the reference genome was built using Bowtie v2.0.6 (Broad Institute, Cambridge, MA, USA); paired-end clean reads were aligned to the reference genome using Tophat v2.0.9 (Broad Institute, Cambridge, MA, USA) [63].

### 4.5. Gene Expression Quantification and Differential Expression Analysis

HTSeq v0.5.4p3 (California Institute of Technology, Pasadena, CA, USA) was used to count the numbers of reads mapped to each gene. Read counts for each gene were normalized to reads per kilobase of exon model per million mapped reads (RPKM), which is currently the most widely used method for estimating gene expression levels [64]. For differential expression analysis of two conditions (two biological replicates per condition), differentially expressed genes between the two conditions were identified with the DESeq package [65]. Genes with an adjusted *p*-value <0.05 identified by DESeq were considered to be “differentially expressed”. Functional classification of the DEGs was conducted with BLAST analysis with the Swiss-prot database or was based on the Rice TIGR release 7.0. The GOseq R package was used to perform GO enrichment analysis of differentially expressed genes, GO terms with corrected *p*-value < 0.05 were considered “significantly enriched” [66]. Kyoto Encyclopedia of Genes and Genomes Pathway (KEGG; http://www.genome.jp/kegg) annotations were assigned according to the KEGG database; we used KOBAS software (Peking University, Beijing, China) to test the enrichment of differentially expressed genes in particular KEGG pathways [67].

### 4.6. Real-Time Quantitative PCR Verification

Based on potential functional importance, eight genes were selected for validation using real-time qPCR. The ∆∆*C*_t_ method was used to evaluate the quantitative variation and actin was used as internal control. All reactions were performed with at least three biological replicates. The sequences of primers used for the qRT-PCR are listed in Table S8.

## Supplementary Materials

Supplementary materials can be found at http://www.mdpi.com/1422-0067/16/05/11398/s1.
