# Supplementary material for: Comparative Analysis of Anther Transcriptome Profiles of Two Different Rice Male Sterile Lines Genotypes under Cold Stress
Source: Int J Mol Sci. 2015 May 18;16(5):11398–416. doi: 10.3390/ijms160511398 (PMC4463707; doi:10.3390/ijms160511398)
Supplement: Supplementary file 1 [file ijms-16-11398-s001.zip › ijms-80031-Supplementary Information/ijms-80031-Supplementary-Figure-for publish.pdf]

Supplementary Information

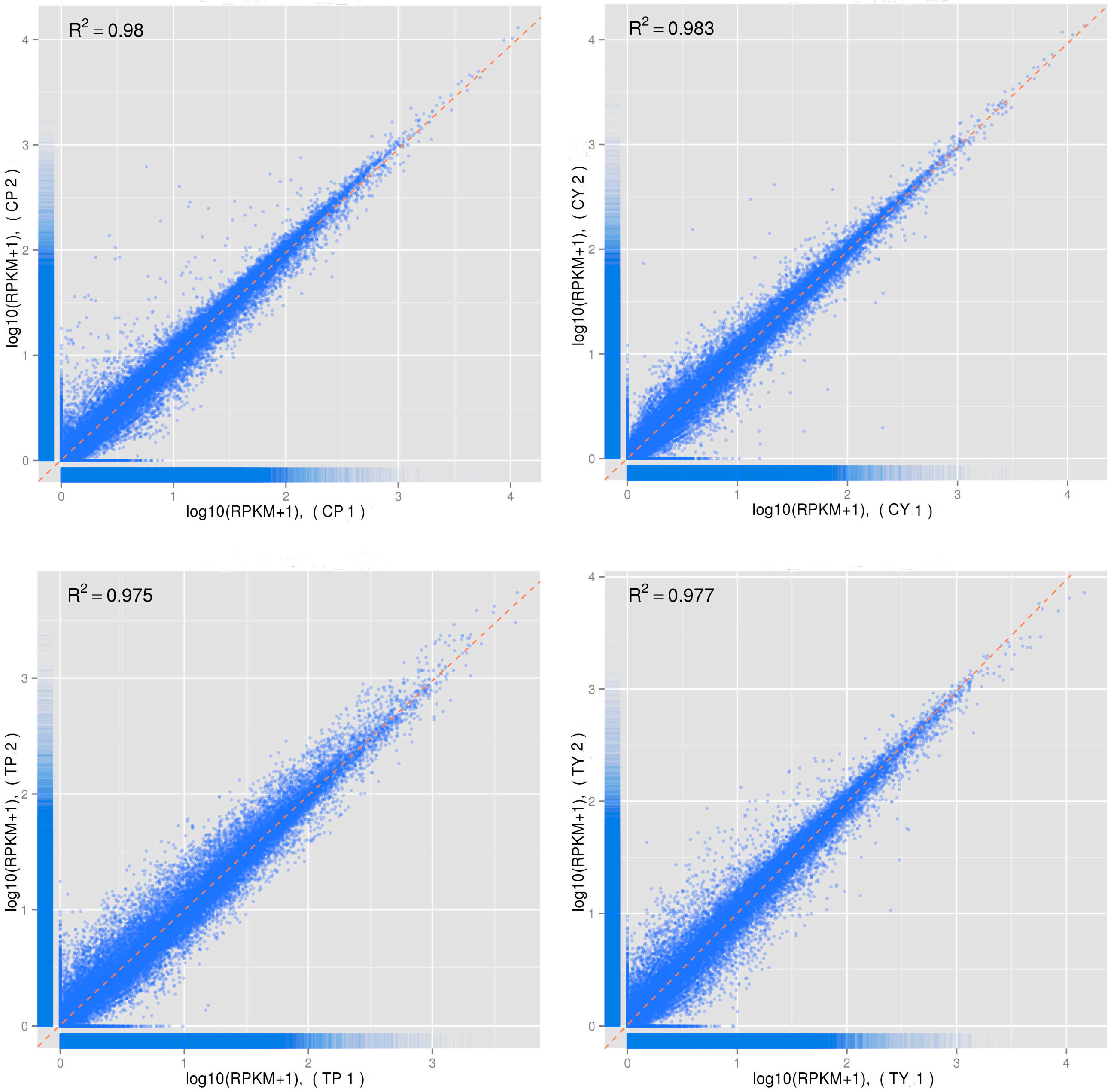

Figure S1. Scatterplots of the Pearson correlation coefficient comparing gene expression scores from biological replicates of each sample. Number 1, 2 denote biological replicates of each sample, respectively.
